# Hot Deformation Behavior and Constitutive Equation of TA15N Titanium Alloy

**DOI:** 10.3390/ma18092067

**Published:** 2025-04-30

**Authors:** Bo Huang, Yang Yu, Wenjun Ye, Songxiao Hui

**Affiliations:** 1 State Key Laboratory of Nonferrous Structural Materials, China GRINM Group Co., Ltd., Beijing 100088, China; hylexb@outlook.com (B.H.); yewenjun@grinm.com (W.Y.); 2 GRIMAT Engineering Institute Co., Ltd., Beijing 101407, China; 3 General Research Institute for Nonferrous Metals, Beijing 100088, China

**Keywords:** hot deformation behavior, constitutive equation, hot processing map

## Abstract

In order to accurately obtain the deformation characteristics and suitable thermal deformation conditions of TA15N titanium alloy and guide the design of deformation process parameters, a Gleeble 1500D was used to conduct hot compression tests on the thermal deformation behavior of a deformed TA15N titanium alloy under the condition of a strain rate of 0.01–10 s^−1^ and a deformation temperature of 850–1090 °C. The constitutive equations for the deformed TA15N titanium alloy based on the Arrhenius formula were developed, and the reliability of the constitutive equations was verified. A thermal processing map of the deformed TA15N titanium alloy was established by using the dynamic materials model (DMM). The research results show that the flow stress of the TA15N alloy decreased with an increase in deformation temperature and a decrease in strain rate. By utilizing electron backscattered diffraction (EBSD), the microstructural evolution and deformation process were analyzed. As the value of η decreased, dynamic recovery (DRV) gradually replaced dynamic recrystallization (DRX). This study supplies a relatively reliable processing interval for the new TA15N titanium alloy.

## 1. Introduction

TA15 alloy is a near-α titanium alloy. Due to its excellent characteristics such as low density, high specific strength, high temperature resistance, and corrosion resistance, it has broad application prospects in the complex working conditions of the oil and gas well drilling industry [1,2,3]. Compared to metallic materials such as steels and aluminum alloys, titanium alloys exhibit greater capabilities in high-temperature plastic deformation—primarily manifested in their high yield-to-tensile ratio—significant deformation resistance, and a pronounced sensitivity in their microstructures to processing parameters [4]. At present, analyzing the hot deformation behavior of an alloy through the construction of constitutive equations and a hot processing map has become a common method for material analysis [5,6,7]. Therefore, exploring the deformation behavior of this titanium alloy by establishing a constitutive equation and hot processing map is the key to ensuring the quality of the material.

Many scholars have conducted relevant research on the deformation behavior of titanium alloys. Xu et al. [8] conducted hot compression experiments on the near-α high-temperature titanium alloy Ti80 in its single-phase region under varying deformation conditions. They found that dynamic recrystallization was the primary mechanism responsible for the flow-softening phenomena in the alloy at lower strain rates (0.01 s^−1^ and 0.1 s^−1^), whereas dynamic recovery dominated at higher strain rates (1 s^−1^ and 10 s^−1^). Peng et al. [9] analyzed the hot deformation behavior of an as-cast Ti60 titanium alloy and established its high-temperature constitutive equation and processing map. Their results indicated that the deformation conditions associated with high-power dissipation efficiency in the processing map corresponded to dynamic recrystallization, with the dynamic recrystallization percentage showing a positive correlation with the deformation temperature. Jha et al. [10] investigated the hot deformation behavior of a Ti-6Al-4V titanium alloy with two distinct microstructural morphologies. Their study revealed that the Widmanstätten microstructure exhibited greater flow softening than the equiaxed microstructure during hot deformation, albeit with a lower deformation activation energy. This phenomenon was attributed to the kinking and shearing of the lamellar α phases, i.e., the instability or dynamic spheroidization processes. Gao et al. [11] studied the flow behaviors and microstructural evolution of three different initial microstructures of a TA15 titanium alloy during hot compression. The results indicated that the stress–strain curve obtained from the duplex microstructure exhibited a higher degree of softening than that obtained from the equiaxed microstructure, but it was lower than that obtained from the Widmanstätten microstructure. This was attributed to the fact that the lamellar α phase bore most of the strain during deformation. Wu et al. [12] investigated the effects of process parameters on the microstructure and texture of a TA15 titanium alloy during hot deformation, analyzing the deformation behavior and microstructural evolution mechanism of the alloy. The results showed that the low-angle grain boundaries (LAGBs) and high-angle grain boundaries (HABGs) were sensitive to the deformation parameters. HAGBs are proportional to strain rate and deformation amount, and they are inversely proportional to deformation temperatures. Due to plastic deformation primarily occurring in the β phase, the texture on the α phase weakens with increasing strain or deformation temperature, while that on the β phase increases. Fan et al. [13] investigated the microstructural evolution of a TA15 titanium alloy during hot working in the single-phase region. The results showed that both the deformation bands and recrystallization consumed deformation-stored energy, which were considered as the softening mechanisms of the alloy, and they existed in a competitive relationship. Specifically, sharper deformation bands could inhibit the nucleation of discontinuous recrystallization, while discontinuous recrystallization reduced the stress concentration, alleviating deformation inhomogeneity and consuming the deformation-stored energy, thereby suppressing the formation of deformation bands. In contrast, the deformation bands with higher morphological complexities exhibit greater subgrain misorientation, which promoted continuous recrystallization in such cases.

Recent research has shown that adding a certain amount of Ni to a titanium alloy can improve its toughness and corrosion resistance, thereby expanding its application prospects in the field of oil and gas drilling [14,15,16,17]. However, there is limited research on the deformation behaviors of new titanium alloys with added Ni elements. In this paper, through hot compression tests, the influence of different forming process parameters on the flow stress of a TA15N titanium alloy during high-temperature deformation is studied. The Arrhenius equation was used to establish a constitutive model for the high-temperature deformation of the TA15N titanium alloy, and the accuracy of the model was verified through a linear correlation coefficient and an average relative error. A hot processing map for the TA15N titanium alloy was established, providing a basis for formulating the reasonable forming processes for the TA15N titanium alloy.

## 2. Materials and Methods

**Figure 1:**
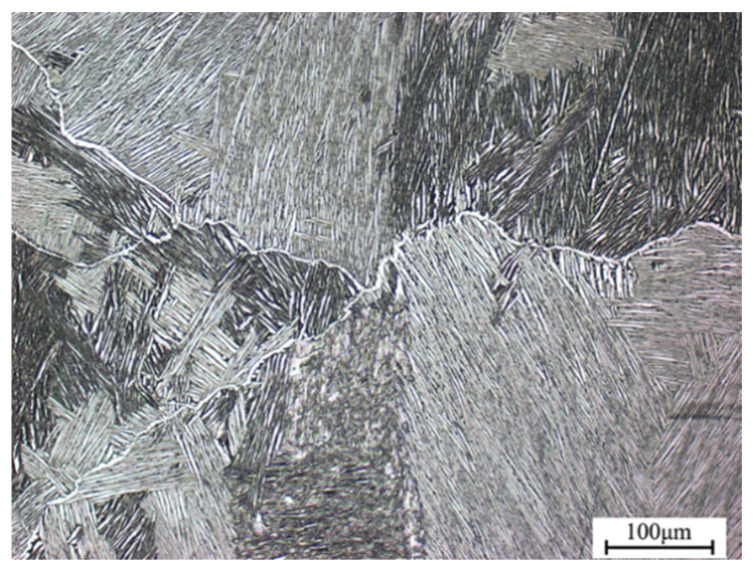
Initial microstructure of TA15N titanium alloy.

The nominal composition of TA15N titanium alloy is Ti-6Al-2Zr-2Mo-2V-0.5Ni. The experimental material was forged and single-phase annealed. The phase transition temperature was determined via the quenching metallography method. When the content of primary α phase in the quenched microstructure was less than 5%, the corresponding quenching temperature was identified as the phase transformation point, which is approximately 970 ± 5 °C. Shown in Figure 1, the initial microstructure is a Widmanstätten structure. The alloy was processed into a cylindrical specimen by wire cutting with a diameter of 8 mm and a length of 12 mm. The hot compression experiments were conducted at strain rates of 0.01–10 s^−1^ and deformation temperatures of 850–1090 °C on a Gleeble 1500D (Dynamic Systems Inc., Poughkeepsie, NY, USA). The experiments were conducted under vacuum conditions. The deformation amount was 60%, corresponding to a true strain value of 0.916. The heating rate was 10 °C/s and the target temperature was maintained for 3 min. To ensure experimental consistency, each experimental condition was repeated at least three times. Immediately after the compression deformation, the specimens were water-cooled to preserve the microstructure.

The compressed sample was cut in the compression direction using a wire cutter. The sample surface was mechanically polished using CMP solution and 80 vol% OPS + 20 vol% H_2_O_2_ solution, respectively. The central area of the deformed sample was observed using a Zeiss Sigma300 field emission scanning electron microscope (Carl Zeiss AG, Oberkochen, Baden-Württemberg, Germany). The EBSD step size was set to 0.1 μm, and the data were processed using the AZtecCrystal (Version 2.1.259) software. Grain boundaries with misorientation angles below 10° were defined as LAGBs, while those with angles above 10° were classified as HAGBs. Metallographic corrosion solution is HF:HNO_3_:H_2_O = 1:3:7 (vol%), and corrosion time is 15 s.

## 3. Results and Discussion

### 3.1. True Stress–True Strain Curves

**Figure 2:**
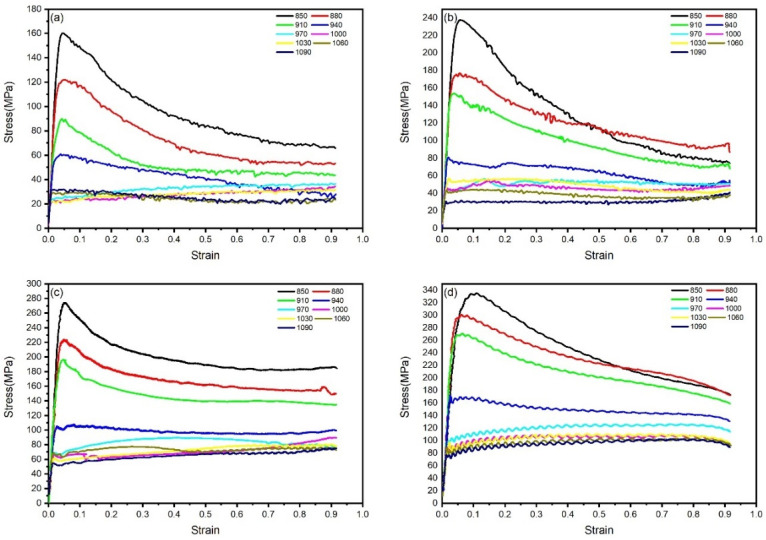
True stress–true strain curves of TA15N titanium alloy with different strain rates and deformation temperatures: (**a**) 0.01 s^−1^; (**b**) 0.1 s^−1^; (**c**) 1 s^−1^; (**d**) 10 s^−1^.

Based on the thermal compression simulation experimental data of TA15N titanium alloy, the true stress–strain curves under different strain rates and deformation temperatures are plotted in Figure 2. It can be observed that, after the start of compression, the stress of TA15N titanium alloy rapidly reaches a peak with the increase in strain, which is a manifestation of work hardening. As the compression continues, the stress begins to slowly decrease and gradually reaches a stable value at the end. In this stage, flow softening gradually increases and reaches a balance with work hardening.

During hot working, DRV and DRX are two crucial flow-softening mechanisms. The manifestation of DRV on the true stress–strain curve is characterized by a gradual increase in flow stress after deformation initiation, followed by a decelerating rate of increase until reaching a steady state. In contrast, DRX typically presents as flow stress first reaching a peak value and then declining to a stable state after deformation commences [18]. As shown in the figure, significant dynamic softening is observed in the alloy when deformed within the temperature range of 850–910 °C, where the flow curves exhibit typical DRX-dominated characteristics. With increasing deformation temperature, the DRX features in the true stress–strain curves gradually diminish. During deformation in the 940–1090 °C range, after reaching the peak stress, no pronounced softening occurs. Instead, a balance between work hardening and dynamic softening is maintained throughout the process, transforming the flow curves into DRV-dominated patterns.

Under the deformation conditions of 880 °C and 0.1 s^−1^, the true stress–strain curve exhibits periodic serrated fluctuations, manifesting discontinuous yielding behavior [19]. Currently, there remains controversy regarding the underlying causes of discontinuous yielding in titanium alloys. Possible deformation mechanisms include: dislocation multiplication theory caused by activation of numerous dislocation sources at high temperatures, the pinning mechanism of Cottrell atmospheres, and high-temperature softening theory of metallic materials [20,21,22,23,24].

By observing the flow curves of materials under different strain rates, it can be seen that the peak stress and steady-state stress of TA15N titanium alloy during hot deformation become higher with the increase in strain rate. As the strain rate increases, the internal dislocation pile-up of the material accelerates, and the material does not have enough time for DRV and DRX. Work hardening becomes dominant and increases the stress value.

### 3.2. Constitutive Modeling

Due to the significant difference in the constitutive relationship between the α phase and β phase of titanium alloy, it is necessary to construct constitutive equations in the two-phase region (α + β) and the single-phase region (β) separately. Currently, the widely used model to characterize the relationship between flow stress and thermodynamic parameters is described by the three forms of Arrhenius functions proposed by Zener et al. [25], which are used to describe the constitutive relationship of alloys. The equations are as follows:

(1) $$Z=\dot{\varepsilon }exp(\frac{Q}{RT})=\left\{\begin{array}{c} A_{1}\sigma^{n_{1}},\;\;\;\;\;\;\;\;\;\;\;\;\;\;\;\;\;\;\;\;\alpha \sigma <0.8\\ A_{2}\exp\left(\beta \sigma \right),\;\;\;\;\;\;\;\;\;\;\alpha \sigma >1.2\\ A{\left[sinh(\alpha \sigma )\right]}^{n}\;\;\;\;\;\;\;\;\;for\;all\;\alpha \sigma \end{array}\right.$$

In the formula, $\dot{\varepsilon }$ represents the strain rate (s^−1^), σ denotes the peak stress (MPa), T signifies the deformation temperature (K), Q indicates the deformation activation energy (J·mol^−1^), and R stands for the gas constant (8.314 J·mol^−1^·K^−1^). α, A_1_, A_2_, A_3_, n_1_, n, and β are material constants independent of temperature, where α = β/n_1_. The constitutive equation presented in this article adopts the form of the hyperbolic sine function.

Taking the logarithm of both sides of the constitutive equation, we obtain:

(2) $$lnZ=ln\dot{\varepsilon }+\frac{Q}{RT}=\left\{\begin{array}{c} {lnA}_{1}+n_{1}ln\sigma \\ {lnA}_{2}+\beta \sigma \\ lnA+n\ln\left[\sinh\left(\alpha \sigma \right)\right] \end{array}\right.$$

From the above formula, we can obtain the relevant constants by performing partial differentiation on the stress term:

(3) $$n_{1}={\left[\frac{\partial ln\dot{\varepsilon }}{\partial ln\sigma }\right]}_{T},\beta ={\left[\frac{\partial ln\dot{\varepsilon }}{\partial \sigma }\right]}_{T},n={\left[\frac{\partial ln\dot{\varepsilon }}{\partial ln\left\lfloor sinh\left(\alpha \sigma \right)\right\rfloor }\right]}_{T}$$

For a certain strain rate, the deformation activation energy can be expressed as follows:

(4) $$Q=R{\left[\frac{\partial ln\dot{\varepsilon }}{\partial ln\left\lfloor sinh\left(\alpha \sigma \right)\right\rfloor }\right]}_{T}{\left[\frac{\partial ln\left\lfloor sinh\left(\alpha \sigma \right)\right\rfloor }{\partial 1000/T}\right]}_{\dot{\varepsilon }}=Rnk$$

**Figure 3:**
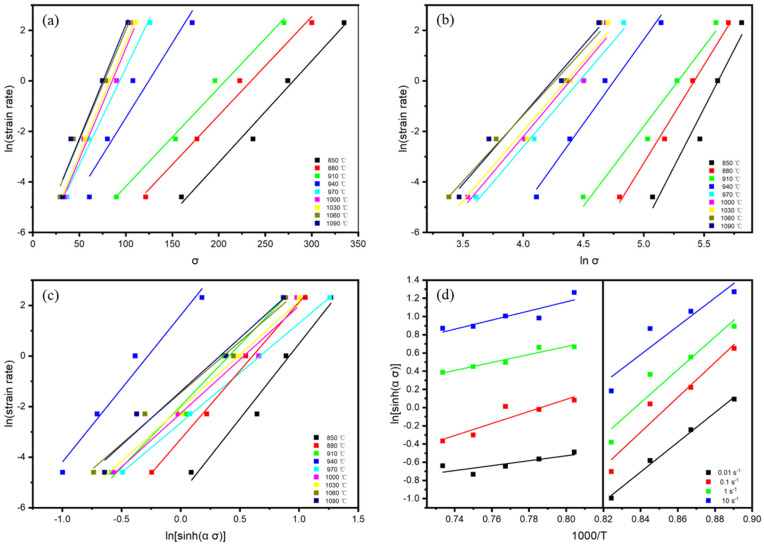
Relation curves between different parameters of TA15N titanium alloy: (**a**) $ln\dot{\varepsilon }$ − σ; (**b**) $ln\dot{\varepsilon }$ − lnσ; (**c**) $ln\dot{\varepsilon }$ − ln[sinh(α σ)]; (**d**) ln[sinh(α σ)] − 1000/T.

As shown in Figure 3a,b, linear fitting was performed on $ln\dot{\varepsilon }$ − lnσ and $ln\dot{\varepsilon }$ − σ at different deformation temperatures. The slopes of the straight lines were calculated and averaged, resulting in n_1_ = 7.5025625 and β = 0.0443825 in the two-phase region and n_1_ = 5.499172 and β = 0.086182 in the single-phase region. From α = β/n_1_, α = 0.005915 in the two-phase region and α = 0.015672 in the single-phase region can be obtained.

Substituting the value of α into ln[sinh(ασ)], linear fitting was performed for ln$\dot{\varepsilon }$–ln[sinh(ασ)] under different deformation temperatures, as shown in Figure 3c. The slopes of the lines were determined and averaged, yielding n = 5.504483 for the two-phase region and n = 4.135173 for the single-phase region. Similarly, linear fitting of ln[sinh(ασ)]–1000/T under different strain rates was conducted, as illustrated in Figure 3d. By calculating and averaging the slopes of the lines and substituting them into the equation, the deformation activation energy was determined as Q = 791.4046 kJ·mol^−1^ for the two-phase region and Q = 160.7561 kJ·mol^−1^ for the single-phase region.

The reported deformation activation energy for the TA15 titanium alloy in the two-phase region ranges from 600–700 kJ/mol [18,26], reflecting its relatively poor deformability in this phase regime. The deformation activation energy observed in this study slightly exceeds this range, which may be attributed to the addition of Ni element. Research indicates that Ni tends to segregate at grain boundaries and phase interfaces, exerting a pinning effect that influences deformation behavior [14,15].

**Figure 4:**
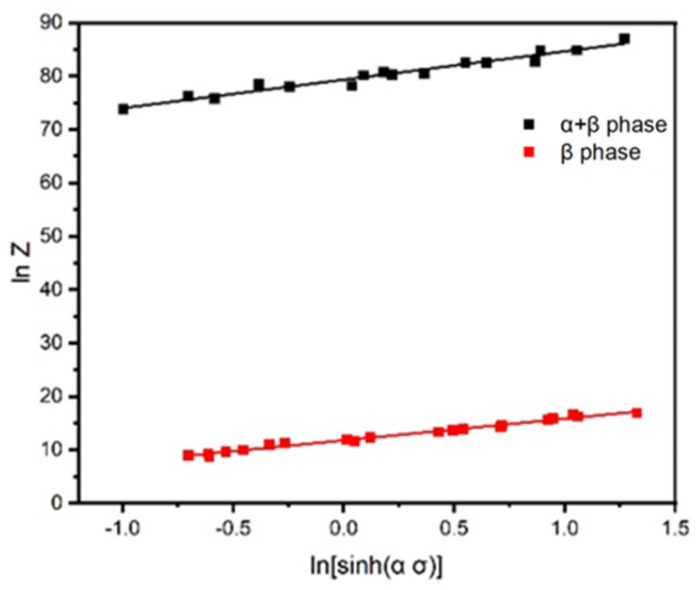
lnZ − ln[sinh(α σ)] curve of Ti65 titanium alloy.

The obtained Q value is substituted into $ln\dot{\varepsilon }$ + Q/RT, and linear fitting is performed on lnZ − ln[sinh(α σ)] for different phase regions. The intercept of the straight line is lnA, as shown in Figure 4. In the two-phase region, lnA = 79.31994, and in the single-phase region, lnA = 11.78349.

Thus, all the parameters involved in the constitutive equation of TA15N titanium alloy were obtained. By substituting these parameters into Equation (1), the constitutive equations of TA15N titanium alloy in both the two-phase region and the single-phase region can be obtained:

(5) $$\dot{\varepsilon }\exp\left(791.4046/RT\right)=e^{79.31994}{\left[\sinh\left(0.005915\sigma \right)\right]}^{5.504483}$$

(6) $$\dot{\varepsilon }\exp\left(160.7561/RT\right)=e^{11.78349}{\left[\sinh\left(0.015672\sigma \right)\right]}^{4.135173}$$

**Figure 5:**
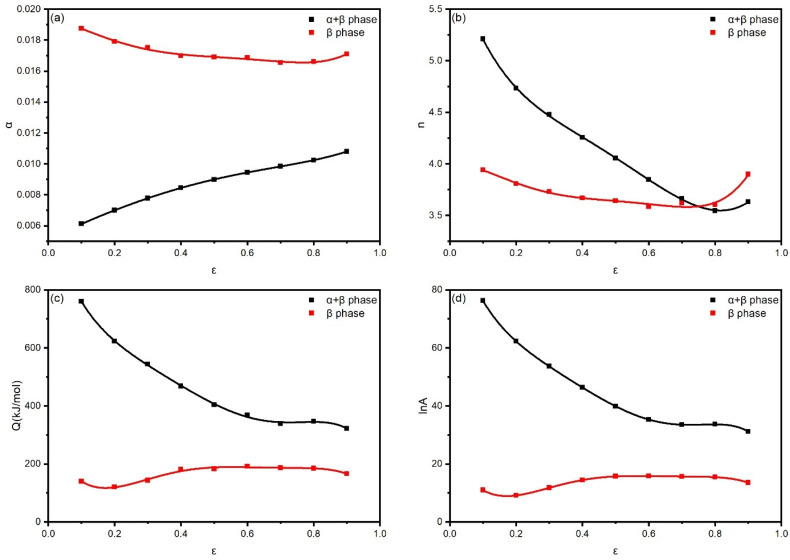
Relationships between strain and material parameters: (**a**) α; (**b**) n; (**c**) Q; (**d**) lnA.

Only peak stress is involved in the calculation of the constitutive equation. However, the influence of strain on material parameters is significant [27]. To comprehensively consider the influence of various factors on the flow stress during hot deformation, the impact of the strain needs further discussion. By calculating, material parameter values of TA15N titanium alloy at true strains ranging from 0.1 to 0.9 were obtained, as shown in Figure 5. It can be seen that the influence of the strain on material parameters cannot be ignored. Research has shown [28] that polynomial fitting can be used to obtain a function of material and strain. By substituting the fitted values into the following formula, the calculated stress values for various strains can be obtained.

(7) $$\sigma =\frac{1}{\alpha }ln\left\{{\left(\frac{Z}{A}\right)}^{\frac{1}{n}}+{\left[{\left(\frac{Z}{A}\right)}^{\frac{2}{n}}+1\right]}^{\frac{1}{2}}\right\}$$

In this article, the material parameters are fitted using a fifth-degree polynomial function, with the following form:

(8) $$P=k_{0}+k_{1}\varepsilon +k_{2}\varepsilon^{2}+k_{3}\varepsilon^{3}+k_{4}\varepsilon^{4}+k_{5}\varepsilon^{5}$$

In the formula, P represents the material parameter and k denotes the polynomial coefficient. The polynomial coefficients corresponding to different material parameters are shown in Table 1 and Table 2.

**Figure 6:**
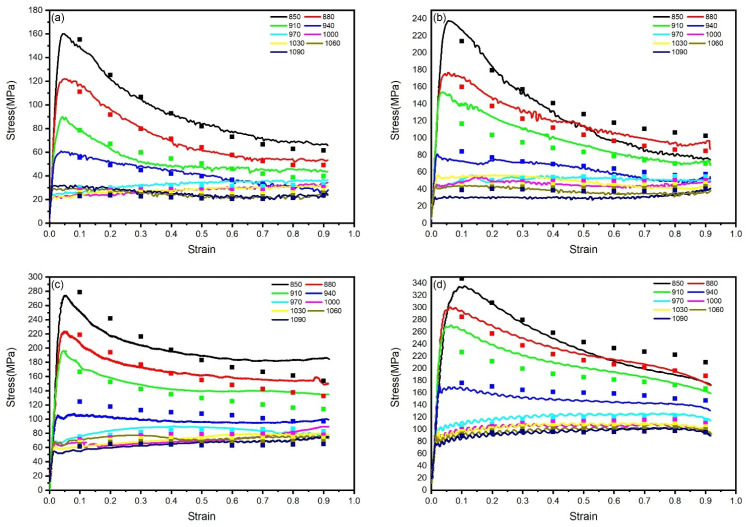
Comparison of the calculated and experimental stress under different strains of the TA15N titanium alloy: (**a**) 0.01 s^−1^; (**b**) 0.1 s^−1^; (**c**) 1 s^−1^; (**d**) 10 s^−1^.

Figure 6 shows a good match between calculated stress and experimental stress, indicating that the Arrhenius model effectively describes the stress of the TA15N titanium alloy. To further verify the accuracy of the constitutive equation, the correlation coefficients (R) and the mean absolute errors (Δ) were used to assess the accuracy of the predictions:

(9) $$R=\frac{\sum_{i=0}^{n}\left(\sigma_{E}-{\bar{\sigma }}_{E}\right)\left(\sigma_{P}-{\bar{\sigma }}_{P}\right)}{\sqrt{\sum_{i=0}^{n}{\left(\sigma_{E}-{\bar{\sigma }}_{E}\right)}^{2}}\sqrt{\sum_{i=0}^{n}{\left(\sigma_{P}-{\bar{\sigma }}_{P}\right)}^{2}}}$$

(10) $$∆=\frac{1}{N}\sum_{i=1}^{N}\left|\frac{\sigma_{E}^{i}-\sigma_{P}^{i}}{\sigma_{E}^{i}}\right|\times 100\%$$

where σ_E_ is the experimental stress; σ_P_ is the predicted stress; ${\bar{\sigma }}_{E}$ and ${\bar{\sigma }}_{P}$ are the average values, and N is the number of data.

**Figure 7:**
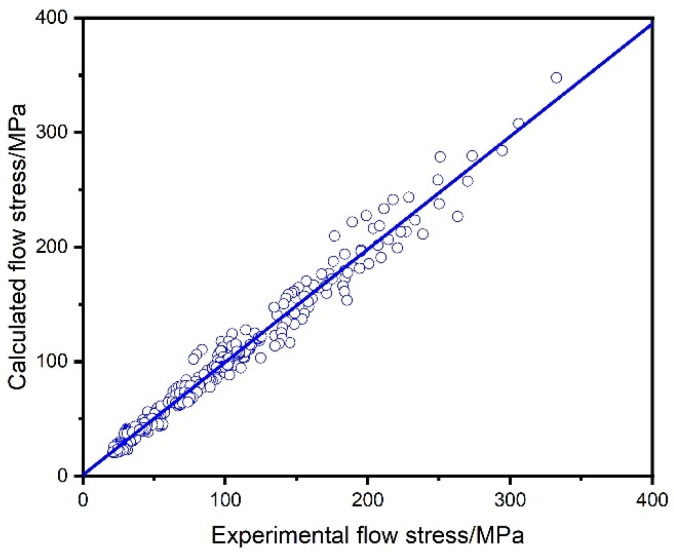
Correlation analysis of flow stress between calculated values and experimental values of TA15N titanium alloy.

As shown in Figure 7, the calculated stress is in good agreement with the actual stress, and the calculated R = 0.988, Δ = 8.04%, indicating that the constitutive equation established has good calculation accuracy

### 3.3. Hot Processing Map

The established constitutive equation of TA15N titanium alloy can determine the relationship between true stress and true strain, but it is not intuitive enough for selecting appropriate thermal processing parameters. Therefore, it is necessary to establish a corresponding hot processing map. Hot processing maps are often used to comprehensively evaluate the processing performance of materials and optimize processing parameters. Based on the dynamic material model, the hot processing map is generated by superimposing the power dissipation efficiency map on the plastic flow instability map. According to the laws of thermodynamics, the total input power P of the system during hot compression can be expressed as:

(11) $$P=\sigma \dot{\varepsilon }=G+J=\int_{0}^{\dot{\varepsilon }}\sigma d\dot{\varepsilon }+\int_{0}^{\sigma }\dot{\varepsilon }d\sigma$$

In the formula, σ represents the peak stress (MPa), G represents the power consumption caused by plastic deformation, while J denotes the power consumption resulting from microstructural evolution. The power dissipation efficiency η is calculated using the following formula:

(12) $$\eta =\frac{J}{J_{max}}=\frac{2m}{m+1}$$

In the formula, m represents the strain rate sensitivity index, which is an important constant for analyzing the deformation behavior and microstructural evolution of titanium alloys. The strain rate sensitivity index m can be calculated using the following formula:

(13) $$m=\frac{dJ}{dG}=\frac{\dot{\varepsilon }d\sigma }{\sigma d\dot{\varepsilon }}={\left[\frac{\partial ln\sigma }{\partial ln\dot{\varepsilon }}\right]}_{\varepsilon ,T}$$

The instability parameter in the instability map is calculated based on different instability criteria. Among them, Prasad [29] proposed the instability criterion based on the principle of large plastic flow irreversible thermodynamics. The calculation formula is as follows:

(14) $$\xi \left(\dot{\varepsilon }\right)=\frac{\partial lg\left[m/\left(m+1\right)\right]}{\partial lg\dot{\varepsilon }}+m$$

In the formula, ξ($\dot{\varepsilon }$) represents the instability factor. When the instability factor is less than 0, flow instability will occur in the alloy.

**Figure 8:**
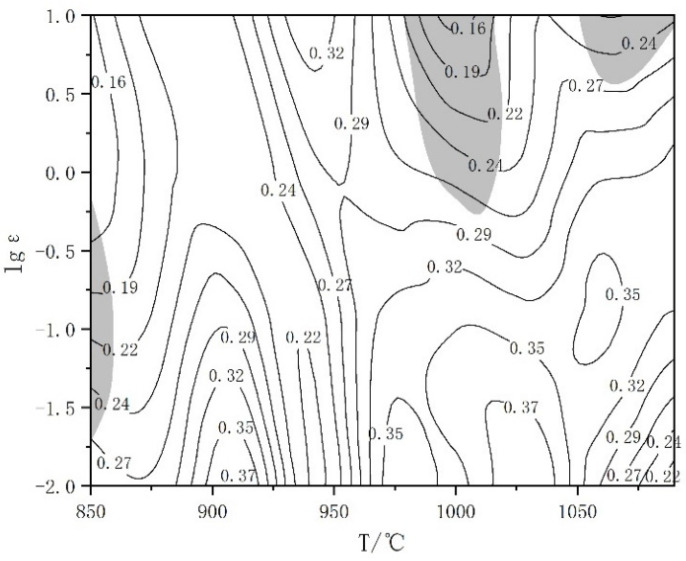
Hot processing map of TA15N titanium alloy.

The hot processing map of TA15N titanium alloy is obtained by superimposing the power dissipation efficiency map and plastic flow instability map, as shown in Figure 8. The contour lines represent the magnitude of the η value, where higher values indicate better hot workability. The material hot processing map is divided into two parts: the instability zone and the safe zone, represented by the gray shaded areas and other areas, respectively. In the instability zone, the energy of material plastic deformation accounts for the majority of the external force input during processing, resulting in low power dissipation efficiency. Therefore, materials in the instability zone are prone to local deformation, forming defects such as adiabatic shear bands and cracks. In the safe zone, the higher the power dissipation efficiency η is, the easier it is for the material to undergo DRX during the hot processing, leading to flow softening and gradually forming stable rheology.

There are three instability zones in Figure 8, where the temperatures and strain rates are 850–860 °C and 0.02–0.75 s^−1^, 980–1020 °C and 0.65–10 s^−1^, 1060–1090 °C and 4.35–10 s^−1^. In these three zones, hot processing of TA15N titanium alloy is prone to cause material deformation instability. Therefore, hot processing in these zones should be avoided in practical production. Near the deformation temperature range of 880–920 °C and 0.01 s^−1^ and 970–1050 °C and 0.01–0.1 s^−1^, the η value is relatively high, indicating that TA15N titanium alloy exhibits good hot processing performance under these deformation conditions.

#### 3.3.1. Metallographic Analysis

**Figure 9:**
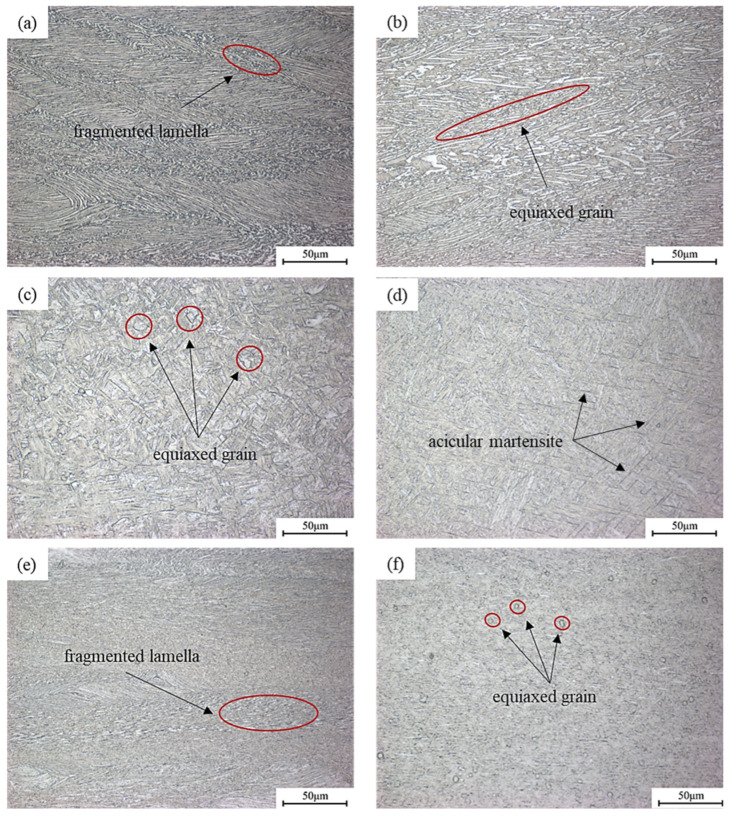
Microstructure of TA15N titanium alloy under different deformation conditions: (**a**) 850 °C, 0.01 s^−1^; (**b**) 910 °C, 0.01 s^−1^; (**c**) 940 °C, 0.01 s^−1^; (**d**) 970 °C, 0.01 s^−1^; (**e**) 850 °C, 10 s^−1^; (**f**) 940 °C, 10 s^−1^.

Figure 9 depicts the microstructure of TA15N titanium alloy under different deformation parameters in the steady-state region, with compression direction being vertical. Under the conditions of strain rate of 0.01 s^−1^ and a deformation temperature of 850 °C, most α lamellar grains elongate, while a small portion of them fragment into fine grains distributed among the lamellae. Due to the low deformation temperature and high deformation resistance, the α lamellar grains tend to be perpendicular to the compression direction but not unified. When the deformation temperature increases to 910 °C, the deformation resistance decreases. A large number of fine equiaxed α grains can be observed, while some α lamellar grains still exist in shorter lengths compared to the lower temperature. It can be seen that, within the range of 850–910 °C, at the same stain rate, the number of equiaxed grains increases with temperature, which also results in an increase in η.

When the temperature rises to 940 °C, the size of equiaxed α grains increases compared to that at 910 °C, indicating that the equiaxed α phase begins to grow at this point, which has a relatively adverse effect on the deformation performance of the alloy. The η value also begins to decrease around this temperature. When the temperature rises to 970 °C, due to the heat generated during deformation, the highest temperature of the alloy during deformation is about 1030 °C and it can be considered to be deformed in the single-phase region. Therefore, the microstructure after water cooling consists of acicular martensite. Since the titanium slip system of the β phase is more than that of the α phase, the deformation capacity of the alloy increases significantly after entering the single-phase region, which is the reason why the η value increases after the temperature exceeds 970 °C.

Compared to a lower strain rate (0.01 s^−1^), the α lamellar grains have no time to elongate and twist at higher strain rate (10 s^−1^), resulting in most of them fragmenting and generating fine grains. The remaining unfragmented α lamellar grains tend towards the vertical compression direction but are not unified. When the deformation temperature was increased to 940 °C, in addition to the fragmented α lamellae, there were also some equiaxed α grains in the microstructure. And at this moment, the value of η is the highest.

**Figure 10:**
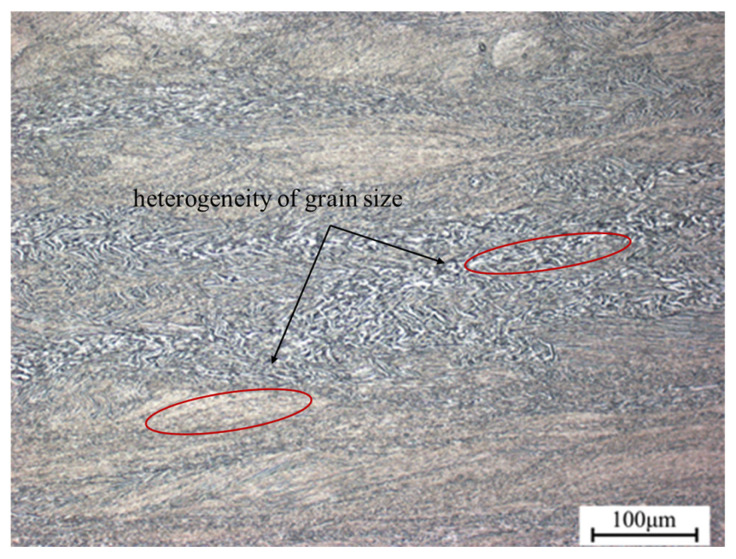
Microstructure of TA15N titanium alloy at 850 °C, 0.01 s^−1^.

Figure 10 depicts the microstructure of TA15N titanium alloy in the instability zone. It is evident that the microstructural changes are uneven. TA15N titanium alloy may exhibit uneven deformation under these conditions. Therefore, processing within the instability zone should be avoided.

#### 3.3.2. EBSD Analysis

**Figure 11:**
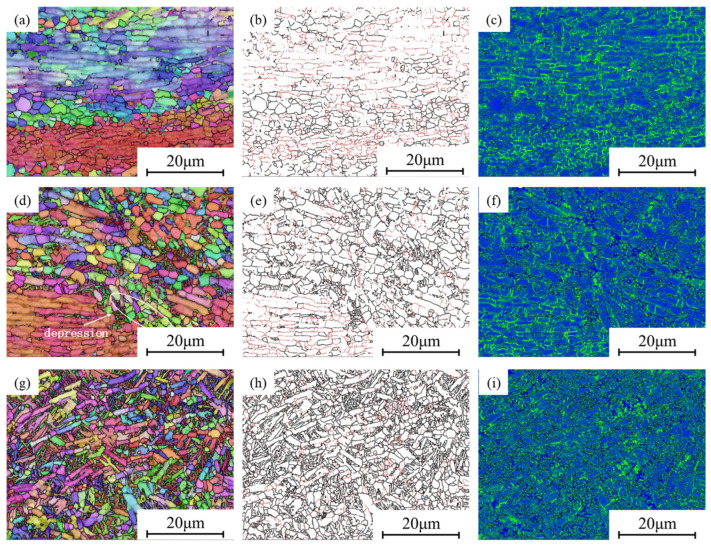
EBSD map of high-η regions: (**a**–**c**) 880 °C, 0.01 s^−1^; (**d**–**f**) 910 °C, 0.01 s^−1^; (**g**–**i**) 910 °C, 0.1 s^−1^.

In Figure 11, EBSD images of stability zones with higher η values (η > 0.25) in the hot processing map, with the compression direction being vertical, are shown. In Figure 11a, it can be seen that most α lamellar grains form deformation bands perpendicular to the compression direction. At the boundaries of different deformation bands, there are equiaxed grains, some of which are relatively coarse. In Figure 11b, HAGBs are represented in black and LAGBs are represented in red, with the contents of the two being 44.7% and 55.3%. Research has shown that DRX can transform LAGBs into HAGBs under appropriate conditions. It can be seen that LAGBs are mainly concentrated within the deformation bands, while they rarely appear in the equiaxed grains. In addition, DRX is related to the dislocation density during hot processing. Dislocation density analysis is usually performed using KAM maps, as shown in Figure 11c. The blue areas have lower KAM values, while the green areas have higher KAM values. It can be seen that the green parts are mainly concentrated near the LAGBs, while the blue parts mainly appear in the DRX grains, and the KAM peak values are lower than those in other areas, indicating that DRX can significantly reduce dislocation density and deformation energy.

As can be seen in Figure 11d, the raised temperature gradually replaces deformed grains with equiaxed grains, and the boundaries of the deformation bands begin to exhibit depressions. This indicates that the degree of DRX at 910 °C is significantly higher compared to that at 880 °C. In Figure 11e, the content of HAGBs is as high as 70.3%, indicating that DRX has been relatively sufficient under this condition, which is also consistent with the conclusions of metallographic analysis. In Figure 11f, not only are there higher KAM values near LAGBs but also higher KAM values near the regions of fine equiaxed grains.

In Figure 11g, the deformation band is basically replaced by equiaxed grains, with only some shorter lamellar grains remaining. In Figure 11h, the HAGB content reaches 85%, which is the highest value within the selected experimental range. Compared with the deformation conditions of 910 °C and 0.01 s^−1^, increasing the deformation rate to 0.1 s^−1^ can provide energy for DRX nucleation, which is beneficial for the refinement of lamellar grains. In Figure 11i, it can be seen that the overall KAM value is relatively low, with only some high KAM values near some LAGBs, indicating that DRX is basically complete at this time.

Clearly, all regions with high η values exhibit varying degrees of DRX. DRX provides uniform and fine grains by eliminating dislocations, thereby resulting in good processability. The deformation should be controlled cautiously to obtain a high-η region.

**Figure 12:**
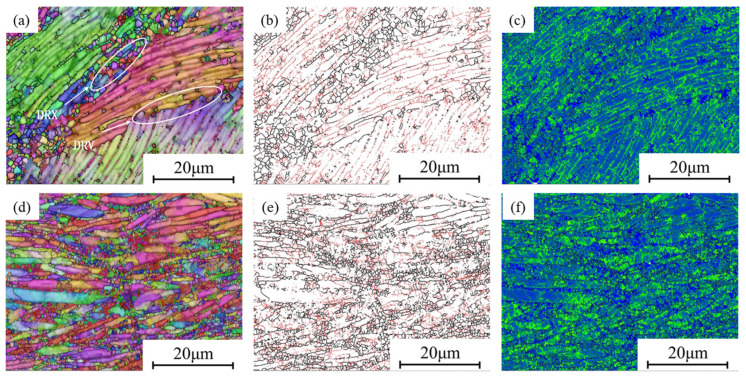
EBSD map of medium-η regions: (**a**–**c**) 880 °C, 0.1 s^−1^; (**d**–**f**) 910 °C, 10 s^−1^.

Figure 12 shows the EBSD image of the region with a medium η value (0.2 < η < 0.25). As can be seen in Figure 12a, α lamellar grains with similar orientations form deformation bands, but these deformation bands are not perpendicular to the compression direction. Some of the deformation bands contain equiaxed grains. On deformation bands without equiaxed grains, their boundaries are straight without depressions. In Figure 12b, LAGBs dominate in this region. These are all characteristics of DRV. In Figure 12c, it can be seen that the KAM value in the DRV region is higher than that in the DRX region.

As shown in Figure 12d, the α lamellar grains are almost entirely perpendicular to the compression direction. However, no deformation bands are formed, and some lamellae are relatively coarse. Distributed among some lamellae unevenly, the α equiaxed grains are relatively fine, indicating that the softening effect of DRX is suppressed. Meanwhile, a higher strain rate also promotes the diffusion of dislocations, resulting in a higher KAM, as shown in Figure 12f.

It can be observed that the softening mechanism of DRX still plays a role in the medium η value region, but DRV gradually replaces DRX as the dominant softening mechanism, resulting in smaller and fewer DRX grains. Compared to the high η value region, the decrease in η value leads to the generation of more dislocations within the material. Furthermore, the microstructure in this region is uneven, which is not conducive to engineering applications.

**Figure 13:**
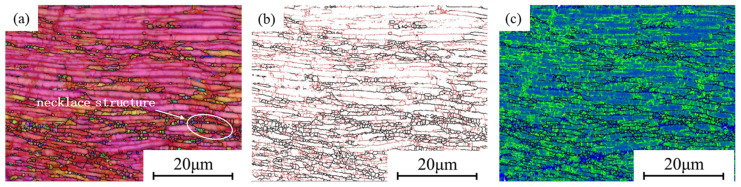
EBSD map of low-η regions: (**a**–**c**) 850 °C, 10 s^−1^.

Figure 13 shows the EBSD image of the low η value region (η < 0.2). It can be observed that most α lamellae are perpendicular to the compression direction and have nearly consistent orientation. Some α lamellae are surrounded by fine equiaxed α grains, forming a necklace structure. This structure is caused by uneven DRX and has adverse effects on the deformation properties. In Figure 13b, it can be seen that there is a high content of LAGBs, accounting for 54.1%. At the same time, due to the high deformation rate, the KAM value in this region is relatively high, as shown in Figure 13c. As the region with the lowest stability in the hot processing map, low η values can lead to unstable plastic flow, resulting in fewer DRX grains and uneven microstructure. Therefore, this region should be avoided during hot processing.

In summary, the deformation mechanism is influenced by the deformation conditions. Within a certain range, increasing temperature is conducive to promoting the occurrence of DRX in TA15N titanium alloy, while increasing the deformation rate will inhibit the occurrence of DRV. Uniform DRX can enable TA15N titanium alloy to achieve a higher power dissipation efficiency η value, which is beneficial to improving processing performance.

## 4. Conclusions

(1) The true stress–strain curves of TA15N titanium alloy exhibit typical DRX characteristics in the lower part of the two-phase region and DRV characteristics in the upper part of the two-phase region and the single-phase region. Increasing the deformation temperature and decreasing the deformation rate are both beneficial to the softening of TA15N titanium alloy, resulting in a decrease in stress.
(2) The constitutive equations were established in each phase region. Comparing the calculated and experimental stress at each strain, it was found that the Arrhenius model can well describe the deformation behavior of TA15N titanium alloy.
(3) The dynamic softening mechanism can be distinguished under different processing conditions, where DRX is primarily observed in the high-η region, and DRV gradually replaces DRX as η decreases. Furthermore, when η is less than 0.2 (low-η region), the necklace structure which is detrimental to deformation will appear.
(4) When forming TA15N titanium alloy, it is advisable to choose a deformation temperature of 880–920 °C and a deformation rate near 0.01 s^−1^, as well as a deformation temperature of 970–1050 °C and a deformation rate of 0.01–0.1 s^−1^ as the thermal processing conditions.

## Figures and Tables

**Table 1:** Polynomial fit coefficients for TA15N titanium alloy material parameters in α + β phase.

	α	n	Q	lnA
k_0_	0.00514	6.06186	1033.649	104.4506
k_1_	0.01032	−11.2505	−3879.81	−399.236
k_2_	−0.00522	31.95226	14,238.57	1465.104
k_3_	0.00292	−50.0756	−31,657.5	−3266.04
k_4_	−0.01004	33.26115	34,302.13	3553.038
k_5_	0.00858	−5.93544	−13,852.8	−1441.04

**Table 2:** Polynomial fit coefficients for TA15N titanium alloy material parameters in β phase.

	α	n	Q	lnA
k_0_	0.01945	4.00375	301.2174	25.78808
k_1_	−0.00507	0.15744	−2764.05	−254.199
k_2_	−0.0328	−11.6607	14,259.47	1314.259
k_3_	0.13879	40.98678	−30,388.1	−2799.63
k_4_	−0.19153	−56.0055	29,404.14	2709.852
k_5_	0.09022	27.20032	−10731.3	−990.804

## Data Availability

The data presented in this study are available on request from the corresponding author due to the involvement of some confidential data from the company.
